# A novel homozygous variant in the *SPG7* gene presenting with childhood optic nerve atrophy

**DOI:** 10.1016/j.ajoc.2022.101400

**Published:** 2022-02-16

**Authors:** Kathrine O. Eriksen, Andreas Reidar Wigers, Iselin Marie Wedding, Anne Kjersti Erichsen, Tuva Barøy, Kristoffer Søberg, Øystein Kalsnes Jørstad

**Affiliations:** a Department of Ophthalmology, Oslo University Hospital, Norway; b Department of Neurology, Oslo University Hospital, Norway; c Department of Medical Genetics, Oslo University Hospital, Norway; d Faculty of Medicine, University of Oslo, Norway

**Keywords:** Optic nerve atrophy, Hereditary optic neuropathy, Hereditary spastic paraplegia, Spastic paraplegia 7, *SPG7* gene

## Abstract

**Purpose:**

To describe a case of hereditary spastic ataxia (HSP) presenting with childhood optic nerve atrophy and report a novel homozygous variant in the *SPG7* gene.

**Observations:**

A 57-year-old man suffering from progressive optic nerve atrophy since childhood eventually underwent genetic testing. A targeted whole exome gene sequencing panel for optic neuropathy identified a novel homozygous variant in the *SPG7* gene, c.2T > G, p.(Met?), which likely abolished production of paraplegin, an inner mitochondrial membrane protein. Subsequent neurologic examination revealed subtle signs of spastic paraplegia and ataxia in keeping with the genetic diagnosis of SPG7.

**Conclusion and importance:**

Spastic paraplegia 7 (SPG7) is an autosomal recessive form of the neurodegenerative disorder HSP. Pure HSP is characterized by spastic paraparesis in the lower limbs, whereas complicated HSP presents additional neurological manifestations. This case report adds to the evidence that SPG7 can present with childhood optic nerve atrophy, preceding the characteristic SPG7 manifestations. SPG7 should be considered in the workup of suspected hereditary optic neuropathy.

## Highlights

• Spastic paraplegia 7 (SPG7) may present with childhood optic nerve atrophy.
• In this case we identified a likely pathogenic, homozygous variant in the *SPG7* gene: c.2T > G, p.(Met1?).
• Workup for suspected hereditary optic neuropathy should include testing for SPG7.

## 1 Introduction

Hereditary spastic paraplegia (HSP) is group of inherited neurodegenerative disorders, characterized by a triad of progressive spastic paraparesis, hypertonic bladder, and mild sensory dysfunction of the lower limbs.^1^ HSP can occur either in a pure or complex form. The latter manifests with additional neurological features, including dementia, mental retardation, ataxia, muscular atrophy, epilepsy, visual dysfunction, peripheral neuropathy, and magnetic resonance imaging (MRI) abnormalities.^1^^,^^2^ HSP displays heterogeneous inheritance, and so far more than 80 genetic types of HSP have been identified.^3^

Spastic paraplegia 7 (SPG7) is an autosomal recessive form of HSP. It is caused by biallelic pathogenic variants in *SPG7*, a gene encoding paraplegin. Paraplegin is a subunit of the ATP-dependent m-AAA protease, an inner mitochondrial membrane protein involved in degradation of misfolded proteins and regulation of ribosome assembly.^4^ *SPG7* maps to Chromosome 16q24.3 and is composed of 17 exons.^5^ About 150 pathogenic *SPG7* variants are described in the Human Gene Mutation Database, ranging from single nucleotide alterations to larger deletions.^6^ A variety of phenotypic features have been reported in complex cases of SPG7, including cerebral and cerebellar atrophy, dysarthria optic neuropathy, cerebellar ataxia, nystagmus, strabismus, blepharoptosis, ophthalmoplegia, motor and sensory neuropathy, amyotrophy, and intellectual disability.^5^^,^^7^, ^8^, ^9^ The onset of SPG7 ranges from 7 to 72 years, but symptoms most commonly present in adulthood.^5^^,^^7^^,^^10^ We report an unusual case of SPG7 presenting with childhood optic nerve atrophy.

## 2 Case report

The patient was a 57-year-old Caucasian male born to first-cousin parents. Since the age of six, he had experienced bilateral progressive vision loss, and he was diagnosed with idiopathic optic nerve atrophy at the age of 20. Prior to referral he had been followed by a general ophthalmologist, who had noted stable visual function of counting fingers for both eyes over the last years. The patient was referred to our section for neuro-ophthalmology because of further acute vision loss for his right eye. He had also experienced two episodes of transient aphasia in the previous months.

The patient's family history was unremarkable. He had neither siblings nor children. He had a past medical history of type 2 diabetes and hypertension. He did not smoke or consume alcohol. His medical treatment compromised antidiabetics (dapagliflozin, metformin, glimepiride), a statin (atorvastatin), and Calcium and Vitamin D supplements. Five years ago, he had experienced an episode of aphasia and convulsions without loss of consciousness. Neurologic workup at that time had included cerebral MRI, computed tomography (CT) angiography, and electroencephalogram, which had been described as normal. A broad-legged gait had been noted on discharge.

**Fig. 1:**
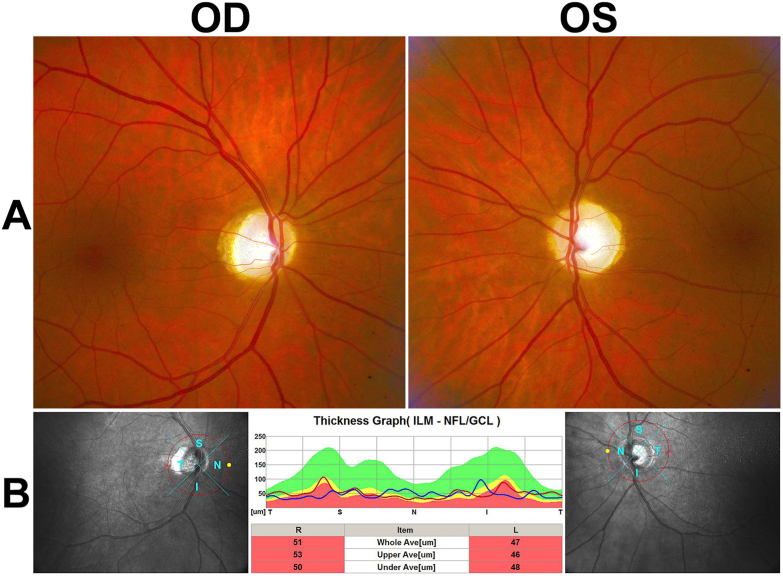
**A.** Fundus photography shows pale optic discs with slightly increased cupping. **B.** Optical coherence tomography of the peripapillary retinal nerve fiber layer displays severe thinning in both eyes.

On ophthalmic examination the visual function was light perception for both eyes. The patient had left esotropia and a conjugate gaze palsy, which was more pronounced vertically than horizontally. He also had pendular nystagmus. There was no blepharoptosis. The intraocular pressure was normal. There was slight cortical cataract. Fundoscopy revealed pale optic discs with slightly increased cupping (Fig. 1A). The funduscopic examination was otherwise unremarkable, without signs of diabetic retinopathy. Optical coherence tomography (OCT) demonstrated severe thinning of the peripapillary retinal nerve fiber layer (mean thickness of 51 μm for the right eye and 47 μm for the left eye; < 99% age-adjusted reference range) (Fig. 1B).

Cerebral CT and MRI were performed to rule out ischemic stroke. The images showed late sequelae of multiple lacunar infarcts and generalized cerebral and cerebellar atrophy. Carotid ultrasound and electrocardiogram were normal. Taken together these findings were consistent with small vessel cerebral disease, and acetylsalicylic acid was added to the patient's medical treatment.

**Fig. 2:**
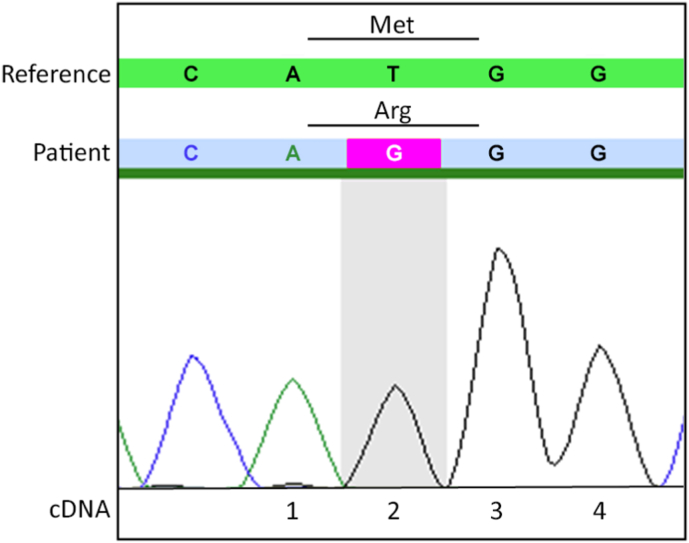
Chromatogram of the Sanger sequencing shows the homozygous c.2T > G variant in *SPG7*. The top green line represents the reference sequence with the ATG start codon encoding methionine, and the bottom blue line represents the patient's sequence, with the AGG codon encoding arginine. (For interpretation of the references to colour in this figure legend, the reader is referred to the Web version of this article.)

A history of bilateral optic nerve atrophy since childhood raised suspicion of hereditary cause, and a genetic screening was performed. Whole exome sequencing with bioinformatical filtration of variants in a gene panel for optic neuropathy identified a novel homozygous variant in *SPG7* (NM_003119.4): c.2T > G, p.(Met?) (chr16:89574827, hg19). The 47 genes in the panel are listed as Supplementary information. The variant was confirmed by Sanger sequencing (Fig. 2). SPG7 c.2T > G alters the start codon and expectedly abolish protein production. The variant is not listed in the Genome Aggregation Database v.2.1.1. However, there are five cases of other heterozygous variants altering p.Met1. In three cases of *SPG7*-related disease, c.1A > T, c.1A > G, and c.3G > A occurred either homozygously or in combination with a known pathogenic variant.^5^^,^^7^^,^^11^ Moreover, two likely loss-of-function variants, c.4delG, p.(Ala2Profs*64) and c.86G > A, p.(W29*), located between p.Met1 and the first downstream in-frame methionine, p.Met44, have been reported. This indicates that p.Met44 cannot be sufficiently utilized as an alternative start codon.^12^^,^ ^13^ Accordingly, c.2T > G, p.(Met?) can be interpreted as likely pathogenic.^14^^,^^15^

The genetic result prompted referral for neurologic evaluation of SPG7. Upon meeting with the neurologist, the patient described unsteadiness and occasional stumbling, which he himself had attributed to visual impairment. The neurological examination revealed several findings: The tongue movements were somewhat reduced. There was mild right-side dysdiadokokinesia. The muscle tone was increased in the lower limbs, and the lower limb reflexes were brisk. Plantar reflexes were indifferent. The muscle strength in the ankle dorsiflexors was mildly reduced. The heel-to-shin test was slightly uncoordinated bilaterally. The gait was broad-based, and the patient was unable to walk on a straight line. Vibration sense was lightly reduced in the lower limbs. Romberg's test was positive. The Scale for the Assessment and Rating of Ataxia (SARA) score was 7.5/40, indicating mild ataxia.^16^ The neurological conclusion was that the medical history and clinical findings were consistent with complex SPG7.

## 3 Discussion

We report the case of a patient with a novel homozygous variant in the *SPG7* gene, c.2T > G, p.(Met1?), presenting with childhood optic nerve atrophy. The patient was first diagnosed as an adult. Neurological work-up revealed subtle spastic ataxia. Accordingly, the final diagnosis was complex SPG7, but apart from optic nerve atrophy, additional neurological manifestations had gone unnoticed until the pathogenic *SPG7* variant was detected.

Hereditary optic neuropathies can occur as an isolated condition (monosymptomatic) or be part of a systemic neurodegenerative disease, in which ophthalmic findings occasionally are the earliest manifestation.^17^ With regard to SPG7, more than 30 cases of optic neuropathy have been described, typically as a feature of complex SPG7.^7^^,^^8^^,^^10^^,^^11^^,^^18^, ^19^, ^20^, ^21^, ^22^ Occasionally, optic neuropathy is subclinical and only evident on OCT.^11^^,^^22^ There are a few reports of childhood-onset optic nerve atrophy in SPG7.^7^^,^^19^^,^^20^ Similarly to our patient, two of these cases presented with progressive visual loss, preceding other SPG7 manifestations by many years.^7^^,^^20^

In the case of our patient, the presenting phenotype was isolated optic nerve atrophy from childhood, and it would take more than fifty years and the discovery of a novel genetic variant to raise suspicion of SPG7. Accordingly, the initial expectation for genetic screening was that the patient could suffer from one of the more common monosymptomatic optic neuropathies, Leber Hereditary Optic Neuropathy (LHON) or autosomal dominant optic atrophy (ADOA), which both can present in childhood. Notably, some ADOA cases later develop other manifestations and are then referred to as ADOA plus syndrome (ADOA+). ADOA + features include ophthaloplegia and ataxia, thereby overlapping the clinical presentation of SPG7 in our patient.^23^ The possibility of dominantly inherited SPG7 with optic nerve atrophy further complicates the phenotypic overlap between ADOA+ and SPG7.^24^ Particularly, seven family members of a French family were initially diagnosed with AODA. Instead of *OPA1* mutations, however, a heterozygous *SPG7* mutation was eventually identified in all affected family members, indicating that SPG7 can also cause dominantly inherited optic neuropathy.^11^ This underscores the value of genetic testing for making the correct diagnosis.

Notably, our patient had previously experienced intermittent neurological symptoms and was referred because of further, acute vision loss for his right eye. Neuroimaging showed ischemic sequelae and generalized cerebral and cerebellar atrophy. In this regard, the involvement of a mitochondrial membrane protein in SPG7 is intriguing; mitochondrial disease may present with acute onset of neurological symptoms and cerebral changes, such as in Mitochondrial Encephalomyopathy with Lactic Acidosis and Stroke-like episodes (MELAS), or acute vision loss, such as in LHON. Still, stroke-like episodes are not considered a clinical feature of SPG7, and we can only speculate as to whether mitochondrial dysfunction predisposed to acute neurological deterioration in our patient.

In conclusion, this case report of a novel variant in the *SPG7* gene adds to the evidence that SPG7 can present with childhood optic nerve atrophy. Our patient displayed phenotypic overlap with ADOA+ in particular, and genetic testing was crucial for making the correct diagnosis. SPG7 should be kept in mind in the workup of suspected hereditary optic neuropathy.

## Funding

No funding was received for this work.

## Intellectual property

We confirm that we have given due consideration to the protection of intellectual property associated with this work and that there are no impediments to publication, including the timing of publication, with respect to intellectual property. In so doing we confirm that we have followed the regulations of our institutions concerning intellectual property.

## Research ethics

We further confirm that any aspect of the work covered in this manuscript that has involved human patients has been conducted with the ethical approval of all relevant bodies and that such approvals are acknowledged within the manuscript.

Written consent to publish potentially identifying information, such as details or the case and photographs, was obtained from the patient(s) or their legal guardian(s).

## Authorship

All listed authors meet the ICMJE criteria.  We attest that all authors contributed significantly to the creation of this manuscript, each having fulfilled criteria as established by the ICMJE.

We confirm that the manuscript has been read and approved by all named authors.

We confirm that the order of authors listed in the manuscript has been approved by all named authors.

## Contact with the editorial office

This author submitted this manuscript using his/her account in EVISE.

We understand that this Corresponding Author is the sole contact for the Editorial process (including EVISE and direct communications with the office). He/she is responsible for communicating with the other authors about progress, submissions of revisions and final approval of proofs.

We confirm that the email address shown below is accessible by the Corresponding Author, is the address to which Corresponding Author's EVISE account is linked, and has been configured to accept email from the editorial office of American Journal of Ophthalmology Case Reports: kathrineoeriksen@gmail.com.

## Patient consent

Consent to publish this case report has been obtained from the patient in writing.

## CRediT authorship contribution statement

**Kathrine O. Eriksen:** Writing – original draft, presentation, Visualization. **Andreas Reidar Wigers:** Conceptualization, Validation. **Iselin Marie Wedding:** Writing – review & editing, Validation. **Anne Kjersti Erichsen:** Validation. **Tuva Barøy:** Writing – review & editing, Validation. **Kristoffer Søberg:** Writing – review & editing, Visualization. **Øystein Kalsnes Jørstad:** Conceptualization, Writing – original draft, presentation, Writing – review & editing, Visualization.

## Conflicts of interest

No conflict of interest exists.
