# Supplementary material for: A novel homozygous variant in the SPG7 gene presenting with childhood optic nerve atrophy
Source: Am J Ophthalmol Case Rep. 2022 Feb 16;26:101400. doi: 10.1016/j.ajoc.2022.101400 (PMC8861420; doi:10.1016/j.ajoc.2022.101400)
Supplement: Multimedia component 2 [file mmc2.docx]

**Supplementary information.** The gene panel included the following genes associated with optic neuropathy:

ACO2, ADAM9, ALDH1A3, ALG13, ALG3, ALMS1, ARHGEF18, ARL13B, ARL2BP, ATXN7, B3GALNT2, B3GALTL, B3GNT1, BFSP1, BLOC1S3, C12orf65, C21orf2, C8orf37, CISD2, DPM1, DPM2, EIF2B2, GJA1, KIF7, LARGE, MFN2, MPDU1, NR2F1, OPA1, OPA3, PAX6, PEX6, PRPS1, RAB18, RAB3GAP1, RAB3GAP2, RTN4IP1, SIX6, SLC25A46, SLC38A8, SOX2, SPG7, STT3B, TBC1D20, TMEM126A, VPS13B and WFS1.
